# Supplementary material for: Are carbon emissions trading and green financial instruments synergistic? -Comprehensive quantitative research based on content analysis
Source: PLoS One. 2024 Mar 7;19(3):e0298601. doi: 10.1371/journal.pone.0298601 (PMC10919601; doi:10.1371/journal.pone.0298601)
Supplement: S1 Data — (ZIP) [file pone.0298601.s001.zip › Data/地级市Z_F工作报告.zip!/╔╜╢1⁄2/═■║ú╩╨/└┤╫╘╣1⁄2╓┌║┼í╛┬φ┐╦╩2╛▌═°í┐_2004.docx]

各位代表：

　　现在，我代表威海市人民政府向大会作政府工作报告，请予审议，并请各位政协委员和其他列席会议的同志提出意见。

　　一、2003年政府工作的回顾

　　过去的一年，在省委、省政府和市委的正确领导下，新一届政府团结和带领全市人民，以“三个代表”重要思想为指导，认真贯彻落实党的十六大和十六届三中全会精神，在历届领导班子打下的良好基础上，解放思想，抢抓机遇，同心同德，干事创业，圆满完成了市十四届人大一次会议确定的各项任务，实现了开好头、起好步的工作目标。

　　（一）切实有效地防止了非典疫情的传入。去年上半年，面对突如其来的非典疫情，各级政府把保护人民群众的身体健康和生命安全放在第一位，以高度的政治责任感，忠实履行第一责任人职责，坚持“防、控、堵、切、群”五字方针，建立了行之有效的领导、防范、监测、信息和应急处理体系，组织全市人民紧急行动，沉着应对，严防死守，杜绝了疫情传入，取得了阶段性重大胜利，保持了威海这方净土。同时，坚持一手抓防治非典，一手抓经济发展，统筹兼顾，科学安排，保持了经济社会发展的良好势头。

　　（二）掀起了新一轮加快发展的热潮。以解放思想为先导，明确了赶超对象，调高了工作目标，建立和完善了考评机制，形成了加快发展的新热潮。全年完成GDP836亿元，比上年增长17.9%，增幅为“九五”以来最高，其中一、二、三产业分别增长4.6%、23.7%和13.2%；财政总收入达到67.2亿元，其中地方财政收入37.2亿元，增长33.4%。在加快发展的同时，人民生活水平明显提高。城乡居民储蓄存款余额达到359.9亿元，增长14.5%；农民人均纯收入4751元，增长7.9%；城镇居民人均可支配收入突破万元大关，增长8.6%。

　　（三）大力实施了对内对外全方位开放战略。整合了园区资源和招商力量，组建了驻韩国办事处，在全省率先启动了网上投资洽谈会和商品交易会，成功举办了韩日招商月活动和一系列国际性会议，实现了招商引资新突破。全市合同利用外资22.1亿美元，比上年增长94.5%；实际利用外资11亿美元，增长82.7%；实际利用内资102.6亿元，增长78%。巩固了传统市场，突出了国际市场开发，对外贸易实现快速增长，完成进出口总值40.3亿美元，增长37.8%，其中出口25.3亿美元，增长31.6%。经济外向度达到40%，提高了6个百分点。

　　（四）加大了固定资产投资和城市建设力度。坚持以大项目带动大投入，以大投入促进大发展，加大了投资强度。全社会共完成固定资产投资370.6亿元，比上年增长119%。实施过千万元的投资项目1085个，其中过亿元的167个，分别增加520个和99个。在城市建设方面，完成了城市发展战略研究和城市三维数字地理空间信息系统开发，制定完善了各类城市规划设计260多项，中心城市实施了世昌大道二期改造和环山路绿化等一批重点工程，荣成、文登、乳山以及部分中心镇也完成了一批基础设施工程，市容市貌有较大改观；引进外资参与了自来水、地下管网、天然气等城市基础设施建设，城市经营迈出实质性步伐。加大了生态市建设力度，不断改善人居环境，创建了全省第一个园林城市群，获得了联合国“人居奖”这一全球改善人居环境领域的最高奖项，极大地提高了威海的美誉度。

　　（五）突出了结构调整这条主线。坚持抓二产带一产促三产，进一步优化了产业结构，三次产业比重达到11：59：30。农业结构调整成效显著，粮经比例达到47：53，林牧渔业产值占农业总产值的78.2%。工业经济实现了速度效益同步大幅增长，规模以上工业实现增加值425亿元、利税110.8亿元，分别比上年增长25.9%和25.8%；完成高新技术产业产值330.4亿元，增长58.1%，占工业总产值的19.7%，提高了5个百分点。现代服务业日益活跃，完成社会消费品零售总额202亿元，增长14.6%，其中，新型业态所占比重达到20%以上。继荣获全国第一个“环保模范城市群”之后，去年我市又成为全国第一个“优秀旅游城市群”。

　　（六）进一步深化了各项改革。坚持以产权制度改革为核心，加快了企业改制步伐，改制面达到85.7%。公司上市取得新的进展，有6家企业上市材料被证监会正式受理，其中2家获准在境内主板上市、2家获准在香港创业板上市。全面停征了乡村公益事业金，农村税费改革不断完善。落实了扶持政策，民营经济得到蓬勃发展，全市个体工商户和私营企业完成增加值230亿元，比上年增长40%；上缴税金4.2亿元，增长31.5%。

　　（七）着力解决影响长远发展的战略问题。坚持立足当前、着眼长远的方针，根据威海的城市定位和产业状况，在总体发展上，确立了建设加工制造业、旅游度假、农副产品出口加工“三大基地”的发展战略；在工业布局上，确立了重点发展运输设备、电子信息、机电工具、轻工纺织服装、食品医药“五大产业群”。为加快制造业基地建设，调整了行政区划，完善了开发区基础设施建设，积极争取、开通和完善了海陆空“三大通道”，新增国际集装箱航线5条，铁路扩能改建工程进展顺利，机场开放工作取得实质进展。围绕清除体制性障碍，理顺了刘公岛管理体制，调整了市区税收征管体制，组建了国资公司和水务集团。以国资公司为平台，向国家开发银行争取了大笔建设资金。以“福”文化为龙头,初步完成了全市旅游业发展总体规划、千公里海岸线旅游规划，确立了刘公岛海权文化主题公园建设思路，启动了威海公园西侧国际会展中心等项目和威海船厂原址开发改造等工程。为促进高新技术产业化和科技型创新企业的成长，在全省首家以市场化运作模式，合资成立了威海创新投资有限公司，并与山大威海分校共建了国际生物技术研究中心，与哈工大（威海）共建了国际微电子研究中心，以此推动高新技术产业的发展。同时，以迁建威海职业技术学院为契机，加大了职业技术教育资源的整合力度，全面提高我市技能型人才的培养能力和水平，应对经济社会快速发展的实际需要。

　　（八）大力促进经济与社会协调发展。全年共组织实施市级以上科技计划291项，取得市级以上重要科技成果76项。理顺了市区高中管理体制，加强了素质教育，推进了“普九”工作，普通高考本科上线率和人口万人比连续10年居全省首位。广泛开展科普教育、爱国卫生、全民健身三项活动，加快推进了卫生、文化、体育、民政等事业改革和发展。深入实施可持续发展战略，开展了土地市场秩序治理整顿工作，加大了污染防治力度，稳定了低生育水平。强化了“双拥”、民兵预备役和国防动员工作，第四次荣获“全国双拥模范城”称号。坚持重心下移，积极开展严打斗争，认真做好人民来信来访工作，维护了社会稳定。依法治市、依法行政进一步推进，精神文明创建活动得到广泛开展。广播电视、新闻出版、民族、宗教、外事、侨务、人防、史志、档案、气象、地震、老龄、残疾人等事业都取得了新的成绩。

　　（九）真心实意为群众办实事。坚持把就业作为民生之本，层层建立目标责任制，将重点援助对象从“4050”扩大到“3545”，全年新增就业4.6万人，转移农村劳动力1.6万人，城镇登记失业率控制在1.7%以内。进一步扩大了社会保险覆盖面，完善了非公有制企业职工及农民工参保办法，城镇职工社会保险覆盖面达到95%以上，企业离退休人员养老金社会化发放率达到100%，农村新型合作医疗制度覆盖面达到39.6%。提高了农村低保标准，分别对1286名和11406名城乡低保对象实施了有效救助。对建筑行业拖欠工资问题进行了清欠。加大了农村公路改造力度，建设乡村公路2000多公里。坚持每年集中办几件与市区居民生产生活息息相关的实事，去年确定的15件，除孙家疃消防站因受土地争议的影响而未能如期完成外，其它均已完成或基本完成。

　　（十）积极转变政府职能。启动了第二轮行政审批制度改革，市级审批事项精简了34.7%，年检事项减少了39.6%。强化了行政审批中心功能，审批事项办结时限比规定平均缩短了30%。健全了人民建议征集、重大事项公示、社会听证、专家咨询等制度，邀请人大代表、政协委员、企业代表和基层群众参加了政府全体会议，开通了政民交流电子信箱，完善了市长公开电话和行风热线，实行了市长和部门主要领导上线制度，共上线200多人次，当场解答问题2100多个，群众满意率达到98%。加强了政府机关廉政建设，严肃查处各类违法违纪案件，政风行风有新的好转。

　　各位代表！开局之年，我市经济发展，社会稳定，政通人和，百业兴旺，迈出了快速稳健、积极有为的一步。取得这样好的成绩，我们倍感鼓舞和自豪，同时也深知这大好局面来之不易。每一个成果的取得，每一项事业的成功，无不凝聚着同志们、朋友们的心血和汗水。在此，我代表市人民政府，向全市所有干部职工、所有建设者、所有纳税人，向对政府工作给予热情支持与监督的人大代表、政协委员、各民主党派、工商联、无党派人士和社会团体，向人民解放军、武警驻威部队指战员和公安干警，向各位老领导、老同志，向所有关心支持威海建设的港澳台同胞、海外侨胞和国际友人，表示衷心的感谢并致以崇高的敬意！

　　总结去年的工作，我们也清醒地认识到存在的困难和问题。主要是：“三农”问题仍是经济社会发展中的薄弱环节，农民增收的任务还很艰巨；工业“群山无峰”的局面并无大的改观，结构性矛盾依然比较突出；旅游资源尚未充分开发和利用，第三产业发展相对滞后；出口产品档次较低，投资结构有待进一步优化；少数群众的生产和生活还比较困难，社会保障体系需要进一步完善；经济与社会发展还有不协调的地方，不稳定的因素仍然存在；政府职能转变还不到位，少数部门和同志思想还不够解放，服务意识不强，存在不正之风和腐败现象等等。同时，今年国家在保持宏观经济政策基本稳定的前提下，将对信贷规模、资金投向、土地管理和财税政策等进行适当调整，对加快发展提出了新的要求，需要我们在工作安排上作出相应的调整和积极的应对。对此，我们将高度重视，进一步增强压力感、紧迫感和责任感，采取切实有力的措施，变困难为机遇，化不利为有利，认真解决存在的矛盾和问题，努力把各方面的工作做得更好，不断夺取现代化建设的新的更大胜利！

　　二、2004年政府工作目标和工作重点

　　今年是深入贯彻落实党的十六大、十六届三中全会精神的重要一年，也是实施“十五”计划的关键一年。做好今年的政府工作，意义重大。展望新的一年，世界范围内产业结构的调整、制造业的转移以及海外人才与科技的回流正呈加速之势，国际经济逐步走向复苏，国内经济进入新的快速增长时期，我们面临着良好的国际国内发展环境和重大的发展机遇；省政府确定的建设胶东半岛制造业基地、山东半岛城市群等一系列重大战略规划将陆续实施，这为我们加快发展注入了新的动力；经过几年的发展，我们积累了许多更快更好发展的物质基础和体制条件，尤其是去年省委、市委加快发展工作会议之后，全市上下比学赶超的热情高涨，干事创业的氛围浓厚，加快发展的后劲充足，有这样好的基础和保证，有全市人民的积极参与和大力支持，我们有能力应对各种竞争和挑战，有信心开创经济和社会发展的新局面！

　　做好今年的政府工作，我们必须以邓小平理论和“三个代表”重要思想为指导，全面贯彻十六大和十六届三中全会精神，按照市十二届党代会二次会议的部署和“实现一个目标、争取两个率先、划分三个阶段、建设三个基地”的要求，坚持全面、协调、可持续发展，紧紧抓住结构调整这条主线，在改革开放、解决“三农”问题、城乡建设、社会事业发展、政府自身建设等重点工作上取得新的进展，促进经济超常规、跳跃式发展，推进社会全面进步，不断提高人民群众的物质文化生活水平，为实现“翻三番”宏伟目标打好坚实的基础。总的目标是：国内生产总值增长16%，力争18%；地方财政收入增长18%，力争20%；全社会固定资产投资增长40%，力争60%；外贸出口总值增长16%，力争20%；实际利用外资增长30%，力争40%；社会消费品零售总额增长13%，力争15%；城镇居民人均可支配收入增长9%，力争10%；农民人均纯收入增长6%，力争7%；城镇登记失业率控制在2%以内；人口自然增长率控制在2‰以内。完成上述目标，工作中必须注意把握以下三条原则：

　　第一，必须坚持发展这个第一要务。发展是解决一切问题的根本途径。要缩小同先进地区的差距，建设全面小康和现代化的新威海，必须打破常规思维，用超常规的措施，谋求跨越式发展。不论外部环境和形势如何变化，加快发展的信心和决心不能动摇。要珍惜来之不易的大好形势，把各方面干事创业的热情和干劲发挥好、保护好、引导好，解决好想干、会干、敢干和干好的问题，继续保持超常规、跳跃式发展的良好势头，在加快发展中不断提升我市的综合实力和竞争能力。

　　第二，必须坚持科学的发展观。坚持全面、协调、可持续的发展观，既是做好政府工作必须长期坚持的重要指导思想，也是解决当前发展中诸多矛盾和问题必须遵循的基本原则。尤其是当前，我们面临着资源和市场的双重约束，发展经济与保护环境的双重压力，国际国内市场的双重挑战，必须按照“五个统筹”的要求，求真务实，脚踏实地，正确处理好速度与质量，规模与效益，当前与长远，经济社会与人口、资源、环境以及改革、发展、稳定的关系，发挥优势，补长短腿，实现经济、社会及生态的良性互动与协调发展。

　　第三，必须坚持“以人为本”的思想。这是我们实践“三个代表”重要思想的具体体现，也是科学决策的价值标准。要把政府工作的出发点和落脚点放在提高人民群众的物质文化生活水平和健康水平上，制定政策、出台措施，都要把握人民利益全面性的要求，切实把人民的利益实现好、维护好、发展好，真正体现“立党为公、执政为民”的本质要求。

　　按照上述要求，新的一年，政府工作要开拓新思路，谋求新发展，打开新局面，必须充分发挥比较优势，搞好重点突破。全方位开放，是我市经济社会发展的重中之重，必须举全市之力，集全市之智，紧紧抓住这个龙头不放松。要坚持以开放促投入，通过招商引资，加大投入力度，优化投资结构，实现多元化融资，建立投资长期增长的内在机制，形成拉动经济增长的新动力；要坚持以开放促改革，在与国际接轨中不断深化市场化取向的改革，突破体制性障碍，促进体制机制创新与转型，不断增强经济发展的活力；要坚持以开放促调整，把扩大开放同产业结构调整以及不同产业内部的结构调整与优化紧密结合起来，继续坚持“抓二产带一产促三产”的基本方针，强化机制、体制和技术创新，形成以现代农业为基础、现代制造业为支撑、现代服务业全面发展的新格局，不断提高经济运行质量和效益；要坚持以开放促其它各项工作的开展，不断吸收先进文明成果，努力改善发展条件，带动经济和社会全面发展。为此，安排今年经济和社会发展的工作重点是：

　　（一）抢抓国际产业转移的有利机遇，构筑更高层次的对内对外全方位开放格局。一是努力保持利用外资高位增长。坚持主攻韩日、扩大港台、开拓欧美，突出打好韩国牌，不断提高利用外资的质量和水平。要重点办好东北亚经济论坛、日韩招商月及赴欧洲、美国、台湾等招商活动，组建驻日本招商办事处，全方位拓宽利用外资的渠道和领域。要扶持办好现有三资企业，促其增资扩股，搞好以商招商。要积极采用跨国并购等新的引资方式，努力实现利用外资方式的新突破。要围绕培育壮大优势产业和拉长产业链，突出制造业、基础设施、农业和服务业招商引资，引进一批辐射带动作用强的大项目。今年全市力争引进过千万美元的外资项目100个以上。二是进一步加大国内招商和对内开放力度。充分发挥威海的城市品牌效应，面向全国各地，面向国有大企业、上市公司、民营企业和各类行业协会，面向大专院校和科研院所，广泛吸引国内资金、技术和人才。抢抓国家实施西部大开发和振兴东北老工业基地战略的机遇，引导企业到内地开辟新的发展空间。组织好赴长三角、珠三角等地的招商活动，吸引那里的外资企业、民营企业来威投资，全年利用内资增长30%以上。三是下大气力办好园区。要按照上级治理整顿土地市场秩序的要求，切实搞好园区的治理整顿。积极采取建设用地指标置换、挖潜盘活存量土地等灵活多样政策和开展土地复垦等措施，拓展经济发展的用地空间。以省级以上开发区为载体，加大投入，完善设施，提高承载能力。制定奖励政策和税收分成办法，加快要素聚集，提高园区土地利用率和单位面积投资率、产出率、利税率。加大对开发区的考核力度，国家级开发区，对外开放主要指标增幅要明显高于全国同类园区，确保每年位次前移；省级开发区，确保实际利用内外资、外贸出口等指标的增幅不低于40%。四是大力开拓国际国内市场。认真研究国家出口退税政策变化所带来的新情况、新问题，综合运用各种贸易方式和手段，努力提高对外贸易的规模和水平。加大出口产品结构调整力度，扩大机电产品和高新技术产品出口比重，大力发展具有更高技术含量的加工贸易。坚持国际、国内两个市场一起抓，引导企业提高产品质量，打造过硬品牌，以质量、品牌和服务巩固市场、开拓市场，不断提高我市产品的国内市场占有率。积极实施“走出去”战略，鼓励有比较优势的企业到国外、市外投资办厂。进一步扩大对外劳务合作，全年外派劳务力争达到4000人以上。五是不断优化投资环境。继续加大对交通、能源等基础设施投入，年内完成威海机场开放改造，继续搞好铁路扩能改造工程，启动威海港三期工程，开工建设威乳高速公路、环海路二期等公路建设项目以及华能威海风力发电、县城电网改造等能源建设项目，提高基础设施配套水平和运营效能。制订和完善为外商服务的具体措施，进一步搞好法制、政策、生活、服务等环境建设，建立健全外商投诉快速反应处理机制，进一步营造亲商、安商、富商的浓厚氛围。

　　（二）把解决好“三农”问题作为重中之重，促进农民增收、农业发展、农村社会全面进步。认真贯彻落实统筹城乡经济社会发展的各项政策，坚持“多予、少取、放活”的方针，采取更直接、更有力、更明确的措施，确保“三农”问题得到进一步解决。一是大力发展特色农业。在加大农田保护力度，稳定粮食生产的基础上，围绕建设农副产品出口加工基地，着力培育花生、果品、蔬菜、药材四大支柱产业；进一步做大奶业，巩固和提高特种动物养殖比重；建设石岛中心渔港码头，加快发展远洋捕捞业，提高养殖业的总体规模和水平，推动精深加工业发展。全年调整经济作物面积20万亩，粮经比达到45：55；远洋渔船达到130艘以上，水产品精深加工比重达到60%以上。二是进一步提高产业化水平。采取扶持政策，培植壮大市级重点农业龙头企业，加快标准化基地建设，扩大农副产品出口。全市以食品加工为主的区域性龙头企业发展到60家以上，农产品出口增长25%以上。三是加大农村投入力度。提高财政支农的比重，加大信贷支农的规模，积极促进农业利用外资，逐步建立政策性农业保险制度，提高水利、农机等农业基础设施建设投资比重。今年新增的教育、卫生、文化等事业经费将主要用于农村。四是不断深化农村改革。全面取消农业特产税，农业税率降低1个百分点。调整粮食补贴政策，把对粮食流通环节的间接补贴改为对种粮农民的直接补贴。进一步稳定土地承包关系，完善土地征用补偿办法，坚决防止和纠正随意征占农民承包地、侵害农民利益的行为。认真开展专项治理，切实减轻农民负担。五是改善农村劳动力转移就业的环境，加快农村城镇化进程。要加强农村劳动力的技能培训，促进农民向城镇有序转移，全年转移农村劳动力1.5万人。把发展农村二三产业、推进农业产业化经营与小城镇建设结合起来，有条件的地方要同步规划城乡基础设施建设，使通讯、信息网络、供电、给排水等逐步向农村延伸覆盖，提高农村的城市化水平。年内要基本完成村村通油路的任务。

　　（三）坚持走新型工业化道路，高质量推进制造业基地建设。紧紧抓住国际制造业转移和建设胶东半岛制造业基地的有利时机，强化区域分工合作，构建产业联盟，加快规模膨胀，推动结构优化升级，全市规模以上工业增加值、利税分别增长18%以上。一是壮大主导产业。重点抓好五大优势产业群：运输设备产业群，主要发展造船、汽车及零部件，构建沿海造船工业带，培植一批汽车零部件生产的小件巨人；电子信息产业群，主要发展微电子产品、通信产品、计算机及外围设备、新型元器件及电子产品、计算机软件“五大系列产品”；机电工具产业群，主要发展机械工具、普通机械、专用设备制造业，增强对外竞争力；轻工纺织服装产业群，主要是上规模创品牌，建成地毯、渔具、服装、抽纱制品出口生产基地；食品医药产业群，主要是以海洋生物为基础，建设一批食品出口加工基地和生物制药基地。五大产业群增加值确保增长20%以上。二是大力培植骨干企业。实施名牌名企战略，加大对32家市级重点企业的扶持力度，引导企业通过收购兼并等方式，加快重组步伐，尽快把企业做大做强。围绕骨干企业的培植，集中力量抓好投资过千万元的工业新建续建项目，全年工业投入确保增长50%以上。进一步加大公司上市工作力度，力争到年底累计有7家公司上市材料上报证监会，6家公司获证监会核准上市，4家公司发行上市，实现融资10亿元以上。三是搞好科技创新，促进高新技术产业发展。坚持用高新技术和先进适用技术改造、提升传统产业，积极发展和引进一批高新技术企业和产品。依托高技术产业开发区和两所驻威高校，建立创业载体，发展高新技术产业带，搞好知识创新基地建设。以威海创新投资有限公司为平台，进一步完善多元化的高新技术投融资机制。大力实施制造业信息化工程，以信息化带动工业化，加快高新技术产业化步伐。全市高新技术产业产值占工业总产值的比重达到22%以上。四是加快企业改革步伐。加大对改制企业的监管力度，健全公有资本有进有退、合理流动的运营机制，确保公有资产保值增值。坚持把企业改革同资产重组、结构调整、招商引资结合起来，加大困难企业、镇村企业改革力度，力争到年底，县属以上企业基本完成改制任务，镇村集体企业改制面由35%提高到60%以上。

　　（四）大力发展旅游业和现代物流业，实现服务业发展的新跨越。按照“一线”、“六板块”的总体规划，以千公里海岸线为依托，以“福”文化为基础，以休闲度假游为主攻方向，高水平、高质量搞好旅游资源和产品开发，抓好荣成福如东海旅游项目、刘公岛海权文化主题公园和空中客车项目、环翠区世界渔村等项目建设，形成威海的新标志、新品牌。发挥旅行社作用，充分利用“人居奖”、“优秀旅游城市群”等金字招牌，加大市场促销力度，大力发展国际旅游，全市旅游总收入、接待海内外游客人数分别增长18%和16%以上。加快综合运输体系、商品市场体系建设，大力发展配送中心、连锁经营、仓储式超市等新的营销方式，新型业态比重达到23%以上。扶持优势企业到农村延伸网点，建立农村消费网络。积极引导居民转变消费观念，开展形式多样的、健康的晚间消费活动。探索实施带薪休假制度，加快培育住房、汽车、文化、健身等新的消费热点，进一步把休闲经济活跃起来。规范中介服务组织，努力摆脱对政府部门的依赖，改进服务方式，提高服务水平。

　　（五）认真做好增收节支工作，进一步提高金融资产质量。坚持依法治税、依率计征、均衡入库，真正把经济发展的成果反映到财政收入上来。大力推进社会综合治税，强化税收源头管理，严厉打击各种偷逃骗税行为，切实做到应收尽收。进一步清理整顿行政事业收费，扩大第二预算管理范围。要加强支出管理，维护预算的严肃性，勤俭节约，杜绝行政管理中的大手大脚和铺张浪费。要以预算编制改革为核心，协调推进国库集中收付、增值税转型等项改革，全面落实出口退税改革措施。加快构建地方公共财政体系，完善市（区）、镇二级财政体制。组织实施预算内投资基建项目“代建制”，建立政府投资后评价制度和责任追究制度，切实提高预算资金的使用效率。

　　积极推进金融改革与发展。要以保安全、保发展为中心，在发展中防范和化解金融风险，不断提高信贷资产质量。合理引导信贷投向，进一步优化信贷结构，优先支持“三农”、重点项目建设以及高新技术产业、骨干企业发展。完善地方性金融组织体系，加快构建开放性地方资本市场。按照上级统一部署，做好农村信用社改革试点工作。加强“诚信威海”建设，加快建立以依法行政和取信于民、服务社会为主要内容的政府信用系统，以信用征信、信用评价、信用自律和失信惩戒为主要内容的企业信用系统，以数据收集、使用和披露为主要内容的个人信用系统，努力形成以道德为支撑、产权为基础、法律为保障的社会信用体系，塑造诚信威海的良好形象。

　　（六）加快城乡建设，进一步优化生态环境。按照“以高新技术产业为主导的生态化海滨城市”的城市定位，站在大威海、山东半岛城市群的高度，以更开阔的视野、更前瞻的眼光，在更高层次上推进城乡建设。一是始终坚持高起点规划。切实把规划作为城乡建设的第一位工作，突出超前性、权威性，敞开城门，引进竞争机制，聘请一流专家和机构进行高起点、高水平规划。年内重点抓好城市总体规划修编、城市空间体系规划、威海至文登城市带概念性规划以及北海旅游度假区旅游发展规划、青岛路两侧城市设计等一批城市详细规划和专业规划的编制工作。二是切实做到高标准建设。强化精品意识，精心策划、设计、施工好每一个建设项目，提高项目的功能质量和艺术含量，提升城市品位。中心城市重点抓好威海公园西侧国际会展中心等项目、威海船厂整体搬迁扩建及原址开发改造、环翠楼公园改造、半月湾开发改造、胶东调水等工程；完成昆明路向西延伸、世昌大道后续改造、光明路至古陌路商业步行街等工程。3个次中心城市和12个中心镇，也要本着增强功能、优化环境、突出特色的思路，规划建设各自的精品工程，带动城镇建设上档升级。三是大力推进生态城市建设。在全社会树立生态环境是“借贷”而不是“继承”的理念，在更高层次上打好环境这张牌。要深入开展争创全省第一个生态市和全国园林城市群活动，加强环保基础设施建设，大力发展循环经济、生态经济和节水型经济，搞好污染防治，巩固和扩大环保模范城市群的成果。重视农业和农村污染的防治。按照中央提出的政策要求，根据我市的实际情况，搞好退耕还林，重点抓好桃威铁路、309国道、威石路等5条主要道路及各市区进出口可视范围内的山坡地绿化，全年完成造林20万亩，植树2200万株，森林覆盖率提高2个百分点。

　　（七）加大扶持力度，加快民营经济和县域经济发展。进一步落实省、市关于加快民营经济发展的各项扶持措施，在市场准入、监督管理、发展政策上与其他所有制企业一视同仁，努力营造一个公平的竞争环境。鼓励民营企业靠大联大，参与公有制企业改制，支持民间资本进入经营性基础设施和公益性事业领域，引导符合条件的民营企业到境内外上市、到境外办企业，参与国际竞争，在更广领域、更大范围内拓展民营经济发展空间。建立健全民营企业信用担保体系、诚信评价体系、人才培训体系、法律服务体系，逐步解决民营企业融资难的问题。广泛开展“双十双百”活动，营造浓厚的发展氛围，全年个体私营经济销售收入增长25%以上。

　　按照发展财源经济的思路和突出特色的原则，促进县域经济发展。有资源优势的地方，要加快由资源开发型向深加工、外向型、科技型转变；有产业基础的地方，要着力培植支柱产业和骨干企业，提高层次，壮大规模；有区位优势的地方，要积极发展配套经济和服务业，把潜在优势转化为现实的发展优势。荣成市依托海洋资源和产业优势，重点发展以海产品工厂化养殖和深加工为主的海洋产业，以船舶和汽车及零配件为主的运输设备制造业。文登市依托现有的工业基础，重点发展机电产品和轻工纺织制造业。乳山市依托矿产资源和农业资源优势，培植以矿产资源和农副产品深加工为主的产业体系。

　　（八）统筹发展各项社会事业，推进精神文明和民主法制建设。一是加快科技创新体系建设，全面提升全市教育水平。强化企业技术创新主体地位，促进产学研紧密结合，提高自主知识产权创新和保护能力。加大对各类创业平台的扶持力度，提高其辐射和带动作用。加快技术交易市场和中介机构建设，增强市场机制对科技资源的配置作用。高质量巩固九年制义务教育，加快普及高中教育，大力发展学前教育、职业教育和成人教育，构建学习型社会。加大教育投入，搞好农村中小学危房改造，加快市区中小学校舍建设。继续搞好校地共建，支持驻威高校发展，并积极创造条件，吸引全国的大专院校、科研院所来威海开展多种形式的合作。

　　二是加紧实施人才强市战略。“国以才立，政以才治，业以才兴”，干任何事情都离不开人。要把促进发展作为人才工作的根本出发点，以能力建设为核心，紧紧抓住培养、引进和用好三个环节，着力建设行政人才、企业经营管理人才和专业技术人才三支队伍。要把事业、待遇、感情作为关键因素，广泛引进高层次人才。要开辟有针对性的教育培训新途径，加大对在职干部职工和农民的培训力度。要以“三个基地”建设的需求为目标，搞好技能型人才、急需外语人才的引进和培养，建立满足经济社会发展需求的梯次人才结构。要制定鼓励人才辈出的政策措施，形成人人都能成才、人人都能为社会发展做出贡献的良好社会氛围。

　　三是积极推进其它社会事业改革与发展。大力培育和弘扬民族精神，加强科普宣传，广泛开展群众性精神文明建设，提高全社会的思想道德水平和文明程度。健全文化和体育市场体系，丰富人民群众的文化和体育生活，积极倡导健康向上的群众文化活动，广泛开展全民健身运动。加强疾病预防控制体系和突发公共卫生事件医疗救治体系建设，提高公共卫生服务水平和应急能力，年内要完成市级传染病医院的建设。坚持把发展经济与控制人口、节约资源、防治污染等项工作一同部署，一并落实，更加自觉地做好人口资源环境工作。积极推进续修新志和依法修志，关心支持妇女、儿童、老龄事业，进一步搞好新闻出版、广播电视、档案、气象、减灾工作，做好民族、宗教、外事、侨务等工作。进一步搞好国防动员工作，加强民兵预备役、“双拥”和优抚安置工作，不断增进军政、军民团结。

　　四是进一步加强民主法制建设。各级政府要自觉接受人大及其常委会的监督，支持人民政协依法履行职责，加强与各民主党派、工商联、无党派人士的联系，办好人大代表建议和政协委员提案，充分发挥工会、共青团、妇联等社会团体的纽带作用。进一步扩大基层民主，搞好社区建设和农村换届选举工作，扩大公民有序的政治参与。要认真实施“四五”普法规划，全面推进依法治市。加强执法监督，强化执法责任制和执法过错追究制。健全完善法律援助体系，依法保障弱势群体的合法权益。要大力加强“平安威海”建设。继续坚持工作重心下移，抓基层、抓基础、抓队伍，进一步强化社会治安综合治理，依法严厉打击各类犯罪活动和“法轮功”等非法邪教组织，扫除“黄、赌、毒”等社会丑恶现象。正确处理新时期人民内部矛盾，继续做好人民来信来访工作，妥善处理群体性事件。牢固树立“安全第一”和“责任重于泰山”的观念，认真落实安全生产的各项规章制度和保障措施，确保一方平安。

　　（九）切实关心群众的生产生活，真心实意地把好事办好、实事办实。从群众最关心、最需要的问题入手，诚心实意地为群众办实事、解难事、做好事。在继续为市区居民办好几件实事的同时，着重抓好以下五个方面：一是努力扩大就业。坚持劳动者自主择业、市场调节就业和政府促进就业的方针，积极开发就业岗位，鼓励企业挖掘潜力分流安置富余人员，支持下岗职工自主创业、自谋职业、灵活就业，年内新增城镇就业4.6万人。要进一步规范企业用工行为，切实维护进城务工农民的合法权益，解决好建筑行业拖欠工程款和工资问题。二是健全社会保障体系。继续做好城市居民最低生活保障工作，确保企业离退休人员养老金按时足额发放，确保失业人员基本生活和及时再就业，建立健全失地农民基本生活保障制度和农村社会养老保险制度。依法扩大社会保险覆盖面，加大社保基金征缴力度，全年参加城镇养老、医疗、工伤保险的人数分别增加2万人以上。要十分重视城市困难家庭住房、子女上学、就医看病等基本生活需求问题以及农村低收入群体尤其是特困家庭、受灾群众的生产生活问题，推动社会救助工作向纵深发展。积极发展残疾人事业，大力推进残疾人的健康、就业、奔小康工作。三是加快发展农村卫生事业。抓好农村卫生院和中心卫生室建设，推行农村新型合作医疗制度，全市农村人口合作医疗覆盖率达到80%以上。四是加快发展社区服务。建立和完善城市社区管理和服务体系，加快服务设施建设，积极开展面向社区居民的家政、养老、托幼、帮困、助残等项服务。五是营造安全放心的消费环境。加大市场监管力度，重点抓好关系群众切身利益的食品、药品市场的专项整治，依法严厉打击制售假冒伪劣商品、走私贩私、非法促销等违法犯罪活动。

　　各位代表！当前，发展处在关键时期，改革处于攻坚阶段，各种矛盾和问题突出而又集中，尤其是在体制创新、利益调整的过程中，必然会产生一些新的困难和矛盾。这些困难和矛盾，由于我们的物质和财力还不够雄厚，还不可能在短期内得到圆满解决，从而会给部分群众的生产生活带来一些不便。但是我们相信，只要我们竭尽全力去做了，就会得到人民群众的充分理解和支持。我们必须懂得人心顺则百事顺、人心稳则社会稳的深刻道理，一定要把改革的力度、发展的速度和社会可承受的程度统一起来，统筹兼顾，协调好方方面面的关系，使政府各项工作真正地体现群众的意愿、符合群众的利益、满足群众的需求。这也是我们一切工作的出发点和落脚点。

　　三、切实加强政府自身建设

　　完成今年的各项任务，各级政府及政府工作人员一定要按照创业、为民、务实、清廉的要求，不断解放思想，更新观念，居安思危，永不自满，始终保持奋发进取、昂扬向上的精神风貌；一定要把学习当成从政为民和工作创新的基本需要，不断提高科学判断形势的能力、驾驭市场经济的能力、应对复杂局面的能力、依法行政的能力和总揽全局的能力；一定要坚持“两个务必”，加强廉政建设，严以律己，廉洁奉公，做到权为民所用、情为民所系、利为民所谋，堂堂正正做人，清清白白为官，扎扎实实办事，经受住党和人民的考验。

　　一要努力建设开明政府。加快政府职能转变，切实使政府从“越位”的地方“退位”，在“缺位”的地方“补位”。进一步搞好市、县两级行政审批中心建设。继续坚持市长及部门主要负责人上线制度，办好行风热线，搞好行风评议，努力为经济社会发展创造公平、宽松的外部环境。《行政许可法》将于今年7月1日正式施行，这对政府的依法行政提出了新的更高的要求，我们一定要牢固树立法治政府是责任政府、有限政府、服务政府、廉洁政府的理念，积极适应《行政许可法》对政府工作的新要求，创新行政管理方式，健全工作制度，完善工作程序，构建行为规范、运转协调、公正透明、廉洁高效的行政管理体制。

　　二要努力建设阳光政府。围绕“事权、财权、人事权”三权，积极推行政务公开，提高政府工作的透明度。进一步扩大政府采购和收支两条线范围，完善人民群众建议征集制度、重大决策事项听证论证制度、评议考核制度、媒体公开制度等，自觉接受群众、社会和舆论监督。要进一步落实人民群众的知情权、参与权、选择权和监督权，继续邀请人大代表、政协委员、企业代表和基层群众参加政府重要会议，使人民群众能够更加充分地发表意见、参政议政。

　　三要努力建设责任政府。要把加快发展的良好愿望与求真务实的科学态度结合起来，把长远规划与分步实施结合起来，把大胆创新与遵纪守法结合起来，既对上级负责又对下级负责，既对当前负责又对长远负责，既对局部负责又对全局负责，严格遵守国家的政策规定，防止和避免出现大的偏差，始终保持正确的前进方向。要坚持从严治政，说实话、办实事、求实效，力戒官僚主义、形式主义，坚决反对欺上瞒下、弄虚作假行为。要树立正确的政绩观，严禁搞劳民伤财的“政绩工程”和“形象工程”。按照省委、省政府的要求和市委的工作部署，市政府确定今年为“工作落实年”，各级政府都要把抓落实作为首要任务，把市委、市政府已经确定的各项发展战略、措施和目标，层层分解，明确任务，明确时限，明确责任，明确奖惩，切实把各项工作落到实处，一抓到底，抓出成效，干出水平。要大力开展行政效能监察，解决行政不作为、乱作为、慢作为和推诿扯皮等问题。坚决制止和严肃查处行政机关滥用权力的问题，对侵犯公民权利、侵害群众利益的人和事，一经发现，要严肃处理；对执法犯法、徇私枉法、欺压百姓的少数害群之马，要坚决绳之以法，绝不姑息!

　　各位代表！新的征程呼唤着我们，新的目标激励着我们，我们一定要紧密团结在以胡锦涛同志为总书记的党中央周围，以邓小平理论和“三个代表”重要思想为指导，在中共威海市委的坚强领导下，解放思想，干事创业，再接再厉，乘胜前进，不断夺取威海改革开放和现代化建设的新胜利！
